# Cumulative live birth rate and neonatal outcomes after early rescue ICSI: a propensity score matching analysis

**DOI:** 10.1093/hropen/hoad046

**Published:** 2023-11-23

**Authors:** Yaping Jiang, Lei Jin, Bo Huang, Li Wu, Xinling Ren, Hui He

**Affiliations:** Reproductive Medicine Center, Tongji Hospital, Tongji Medicine College, Huazhong University of Science and Technology, Wuhan, People’s Republic of China; Reproductive Medicine Center, Tongji Hospital, Tongji Medicine College, Huazhong University of Science and Technology, Wuhan, People’s Republic of China; Reproductive Medicine Center, Tongji Hospital, Tongji Medicine College, Huazhong University of Science and Technology, Wuhan, People’s Republic of China; Reproductive Medicine Center, Tongji Hospital, Tongji Medicine College, Huazhong University of Science and Technology, Wuhan, People’s Republic of China; Reproductive Medicine Center, Tongji Hospital, Tongji Medicine College, Huazhong University of Science and Technology, Wuhan, People’s Republic of China; Reproductive Medicine Center, Tongji Hospital, Tongji Medicine College, Huazhong University of Science and Technology, Wuhan, People’s Republic of China

**Keywords:** early rescue ICSI, conventional ICSI, pregnancy outcomes, neonatal outcomes, clinical outcomes, short insemination, total fertilization failure, IVF, cumulative live birth rate, propensity score matching

## Abstract

**STUDY QUESTION:**

Is early rescue ICSI (E-RICSI) an effective and safe technique compared to conventional ICSI?

**SUMMARY ANSWER:**

Despite the higher multi-pronucleus (PN) rate compared to conventional ICSI, E-RICSI did not add extra risks to clinical and neonatal outcomes.

**WHAT IS KNOWN ALREADY:**

Based on the finding that the second polar body was released in 80% of fertilized oocytes by 4 h after exposure to spermatozoa and in ∼90% of fertilized oocytes by 6 h, E-RICSI brings forward the timing of rescue ICSI to 6 h after initial insemination, and effectively prevents oocyte aging and embryo-uterus asynchrony. However, some researchers still voice concerns about the efficacy and safety of E-RICSI, and comparative studies are limited.

**STUDY DESIGN, SIZE, DURATION:**

A retrospective cohort study was conducted on patients who underwent conventional ICSI or E-RICSI treatment between January 2015 and December 2020 at a university-affiliated hospital. Using 1:1 propensity score matching, 1496 cases entered each group.

**PARTICIPANTS/MATERIALS, SETTING, METHODS:**

In total, 1496 couples undergoing conventional ICSI oocyte retrieval cycles and 1496 undergoing E-RICSI oocyte retrieval cycles were enrolled in this study, and basic clinical characteristics, embryologic data, clinical outcomes and neonatal data were compared between groups. The embryos in the E-RICSI group were divided into two subgroups: those fertilized by iIVF (IVF subgroup) and those fertilized by E-RICSI (E-RICSI subgroup); the embryologic data, clinical outcomes, and neonatal data for these subgroups were also compared with the conventional ICSI group. Logistic regression was used for statistical analysis with potential confounder adjustment.

**MAIN RESULTS AND THE ROLE OF CHANCE:**

The 2PN rate, blastocyst formation rate, and viable blastocyst formation rate of the E-RICSI group were significantly lower compared to the conventional ICSI group (2PN rate: *P* < 0.001; blastocyst formation rate: *P* < 0.001; viable blastocyst formation rate: *P* = 0.004), and the multi-PN rate in the E-RICSI group was significantly higher than the conventional ICSI group (*P* < 0.001). However, the number of 2PN embryos, normal cleavage embryo rate, Day 3 high-quality cleavage embryo rate, and high-quality blastocyst rate were similar between groups. When considering the IVF embryos and E-RCSI embryos in the E-RICSI group independently, the 2PN rate of the conventional ICSI group was significantly lower than E-RICSI subgroup but higher than the IVF subgroup, whereas the blastocyst formation rate and viable blastocyst formation rate were higher than E-RICSI embryos but comparable to IVF embryos. As for the clinical and neonatal outcomes, the implantation rate of the E-RICSI subgroup was significantly lower than the IVF subgroup but comparable to the conventional ICSI group, while the low birthweight (LBW) rate was significantly lower compared with the conventional ICSI group but similar with the IVF subgroup. No other differences were observed among the three groups for cumulative clinical pregnancy rate, cumulative live birth rate, and the pregnancy outcomes per transfer including clinical pregnancy, ectopic pregnancy, miscarriage, and live birth, either in fresh or frozen embryo transfer cycles. Furthermore, neonatal outcomes, including cesarean section, sex ratio, LBW, preterm birth, and macrosomia, were similar among groups.

**LIMITATIONS, REASONS FOR CAUTION:**

This study is limited by the retrospective design, limited sample size, and short follow-up period. However, our study underlies the need for large-scale, multi-center randomized controlled trials with long-term follow-up.

**WIDER IMPLICATIONS OF THE FINDINGS:**

Short-term insemination (3 h) combined with E-RICSI may be a safe and effective method to prevent the occurrence of total fertilization failure, and patients with normal or borderline sperm could be encouraged to try IVF first.

**STUDY FUNDING/COMPETING INTEREST(S):**

This study was supported by grants from the National Key & Development Program of China (No. 2021YFC2700603) and the National Natural Science Foundation of China (No. 81801443). The authors declare no conflicts of interest.

**TRIAL REGISTRATION NUMBER:**

N/A.

WHAT DOES THIS MEAN FOR PATIENTS? Infertile couples often undergo IVF treatment, where eggs (oocytes) are incubated with sperm outside the body to achieve fertilization, i.e. the formation of an embryo. If IVF is unsuccessful (total fertilization failure: TFF) and no embryos are formed, intracytoplasmic sperm injection (ICSI) can be carried out, when a single sperm is injected directly into the oocyte. For couples where IVF results in TFF, the same oocytes that underwent IVF may be injected with sperm—a technique called ‘rescue ICSI’—which involves a delay in achieving fertilization but is usually successful. This delay, which may last many hours, is thought to have negative effects on the oocyte. Therefore, in our study, we investigated whether reducing this delay would result in the birth of healthy babies. The technique tested is called ‘early rescue’ ICSI (E-RICSI), which brings forward the timing of rescue ICSI to 4–6 h after insemination (IVF). Theoretically speaking, E-RICSI can prevent oocyte aging/damage and reduce the unbalanced development of embryo and uterus caused by the delay, as well as decrease the rate of TFF during IVF treatment. However, both patients and doctors are often concerned about the efficacy (ability to produce the desired result) and safety of E-RICSI.

In this study, we compared the results from almost 3000 patients who had been treated with E-RICSI or conventional ICSI (i.e. no prior IVF), in terms of embryo characteristics, number of live births, overall health of the baby, and other relevant outcomes. Cumulative live birth rate was the main outcome analyzed because it includes the live births from both fresh and frozen cycles and is a better indicator of overall success of a treatment. Neonatal outcomes in both fresh and frozen cycles were also evaluated. Based on these results, the conclusion was that E-RICSI did not add extra risks to clinical and neonatal outcomes; however, these findings must be replicated in large-scale, multi-center trials with long-term follow-up of the offspring.

## Introduction

IVF and ICSI are currently the most commonly used forms of ART. However, the application of ICSI has dramatically increased over the past few decades. According to data from the National Assisted Reproductive Technology Surveillance System (NASS) in the USA, the proportion of ICSI cycles increased from 36.4% in 1996 to 76.2% in 2012, and the use of ICSI among patients with non-male factor infertility had risen 3-fold during this period (Boulet *et al.*, 2015). These figures suggest a shift toward increased ICSI use instead of conventional IVF for patients with borderline or even normal semen characteristics. The desire to avoid the risks of total fertilization failure (TFF) or low fertilization in conventional IVF and increase the likelihood of pregnancy may be responsible for this conversion.

TFF occurs in 3.5–20% of conventional IVF cycles (Liu and Baker, 2000; Bhattacharya *et al.*, 2001; Krog *et al.*, 2015), and most of the cases are caused by the failure of sperm to penetrate the oocyte (Mahutte and Arici, 2003; Beck-Fruchter *et al.*, 2014). ICSI bypasses natural barriers to fertilization by injecting the sperm into the oocytes and is regarded as a solution for TFF or low fertilization in conventional IVF. However, the TFF and low fertilization in conventional IVF is difficult to predict (Mahutte and Arici, 2003), and there is insufficient evidence to support the use of ICSI over conventional IVF for all patients, especially for those with non-male factor infertility (Boulet *et al.*, 2015; Haddad *et al.*, 2021). Excessive use of ICSI does not appear to be an effective method to increase the probability of pregnancy and live birth, and there has been some controversy regarding the health of children born after ICSI. Some researchers have expressed their concerns regarding a possible increased risk of chromosomal abnormalities (Gjerris *et al.*, 2008; Belva *et al.*, 2020), imprinting disorders (Allen and Reardon, 2005; Amor and Halliday, 2008), congenital malformations (Qin *et al.*, 2015), and delayed psychological and neurological development (Sandin *et al.*, 2013) following ICSI compared with naturally conceived children, while other researchers believe that the observed adverse outcomes are attributable to parental factors, not the ICSI technique, and numerous studies have confirmed the health of children born after ICSI (Fauser *et al.*, 2014; Esteves *et al.*, 2018). In recent years, short-time insemination combined with early rescue ICSI (E-RICSI) has been proposed to prevent TFF and low fertilization in conventional IVF (He *et al.*, 2018).

Rescue ICSI (RICSI), defined as the reinsemination of the failed-fertilized oocytes by ICSI after conventional IVF (Nagy *et al.*, 1993; Chen and Kattera, 2003), is usually implemented after the fertilization states of oocytes are confirmed. According to the Istanbul consensus (Alpha Scientists in Reproductive Medicine and ESHRE Special Interest Group of Embryology, 2011), the fertilization state is determined by the presence of pronuclei (PN) and a second polar body at 16–18 h after insemination. Therefore, the RICSI has to be performed on Day 1 after oocyte retrieval, commonly known as ‘late RICSI (L-RICSI)’. However, owing to the oocytes aging and embryo–uterus asynchrony, the fertilization rate of L-RICSI ranges from 28.0% to 60.2%, and pregnancy outcomes of L-RICSI range from 0% to 20.7%, which is consistently disappointing (Yuzpe *et al.*, 2000; Lombardi *et al.*, 2003; Sermondade *et al.*, 2010). Then, several studies have demonstrated that the second polar body is released in 80% of fertilized oocytes by 4 h after exposure to spermatozoa and in ∼90% of fertilized oocytes by 6 h (Nagy *et al.*, 1994; Payne *et al.*, 1997), indicating that the second polar body is an early indicator of whether the oocyte is fertilized or not. Based on this finding, Chen and Kattera firstly brought forward the timing of RICSI to 6 h after insemination and obtained a normal fertilization rate of 70.3% and pregnancy rate of 48.0%, a technique termed ‘E-RICSI’ (Chen and Kattera, 2003). Owing to the good fertilization rates and pregnancy outcomes (Liu *et al.*, 2014; Huang *et al.*, 2015), E-RICSI has been regarded as insurance to prevent the occurrence of TFF in conventional IVF treatment, encouraging patients with borderline or even normal semen to try conventional IVF in their first cycle.

However, some researchers still have concerns about E-RICSI. In E-RICSI treatment, the unfertilized IVF oocytes have been incubated with semen for several hours, and the removal of granulosa cells around the oocytes and injection of oocytes occur much later than in conventional ICSI (Tesarik, 2003; Chen *et al.*, 2014). Whether these procedures have adverse effects on pregnancy outcomes and offspring’s health is still uncertain, and such studies are limited. Several studies have reported the pregnancy and neonatal outcomes of E-RICSI and find that the pregnancy rate and neonatal outcomes of E-RICSI are comparable with conventional ICSI (Chen *et al.*, 2014; Huang *et al.*, 2015; Xiong *et al.*, 2020; Zeng *et al.*, 2022). However, almost all studies only report the pregnancy outcomes in fresh embryo transfer (ET) cycles, and the pregnancy outcomes in subsequent embryo thawing cycles are not counted. With the advances in embryo freezing and thawing techniques and the advocation of ‘freeze-all’ policy (Roque, 2015), only reporting pregnancy outcomes in fresh ET cycles is not sufficient for us to evaluate the efficiency of E-RICSI. Cumulative live birth rate (CLBR) has been suggested as a suitable way of reporting IVF success rate (Maheshwari *et al.*, 2015), which incorporates fresh as well as thawed frozen ET cycles, but none of the previous studies have reported this. Furthermore, most previous studies have neglected the differences in sperm quality and source between patients receiving E-RICSI versus conventional ICSI treatment. Furthermore, the sample sizes of previous studies are limited. A well-designed study with a larger sample size and appropriate outcome measure is needed to assess the efficiency and safety of E-RICSI.

Therefore, in order to evaluate the efficiency and safety of the E-RICSI technique comprehensively, we retrospectively analyzed the CLBR and other clinical and neonatal outcomes of patients undergoing E-RICSI and conventional ICSI treatment in our center. Meanwhile, to minimize selection bias and balance population characteristics, propensity score matching (PSM) was used to select appropriate controls.

## Materials and methods

### Study design

This retrospective study enrolled all patients who underwent conventional ICSI or E-RICSI treatment between January 2015 and December 2020 at the Reproductive Medicine Center of Tongji Hospital. We excluded patients who met the following criteria to minimize the impact of sperm quality and other confounding factors: using frozen oocytes, IVM, or artificial oocyte activation; surgical retrieval of sperm; preimplantation genetic testing cycles; and severe oligospermia or azoospermia (percentage of forward motile sperm <10% or sperm concentration <5 × 10^6^/ml).

**Figure 1.**
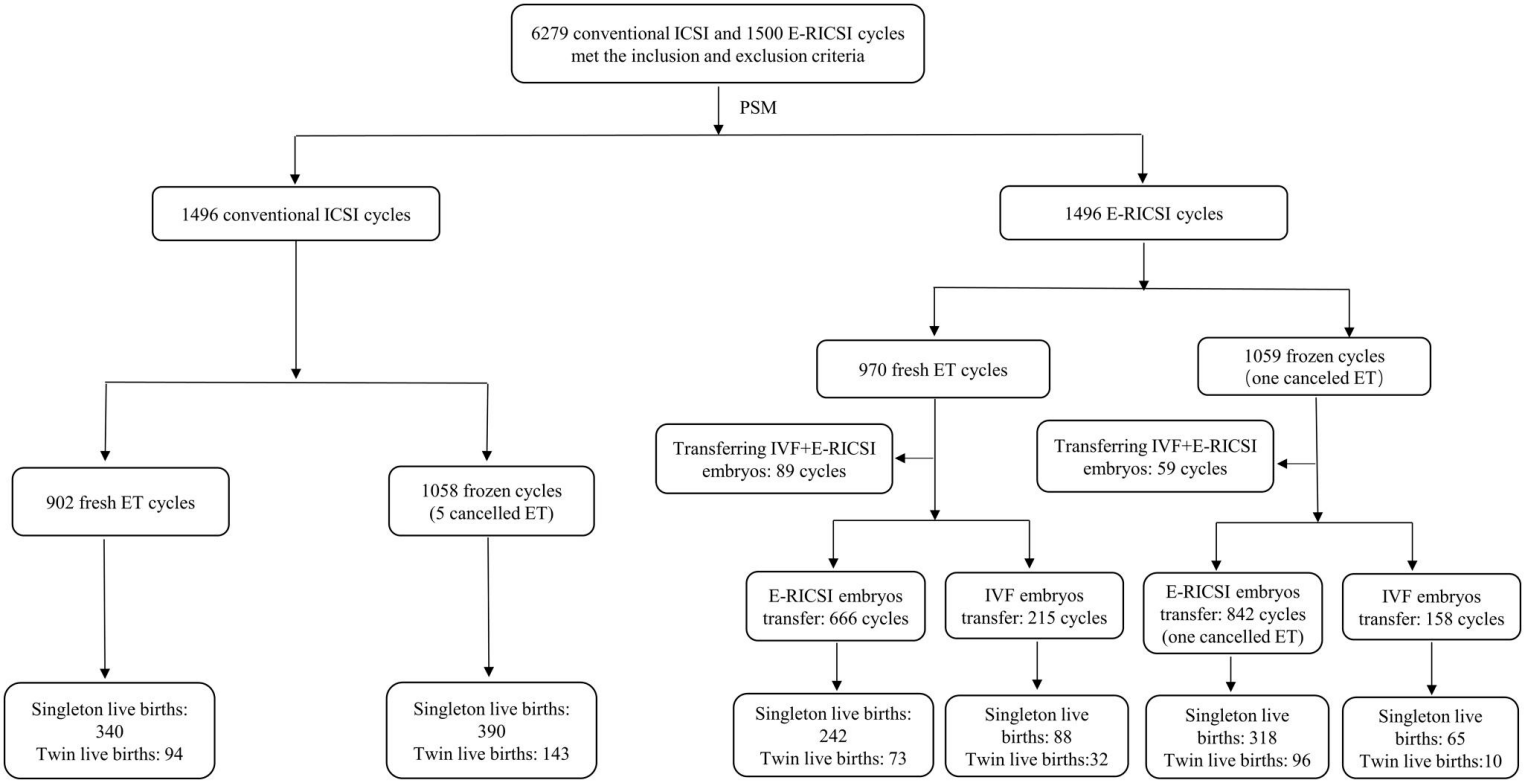
**The workflow chart of patient recruitment in a study of cumulative live birth rate and neonatal outcomes after early rescue ICSI.** E-RICSI, early rescue ICSI; ET, embryo transfer.

The study design is described in Fig. 1. In total, 6279 conventional ICSI and 1500 E-RICSI oocyte retrieval cycles met the inclusion and exclusion criteria and were enrolled in this study. Then, PSM was used to control for confounding variables and minimize selection bias. As a result, 1496 conventional ICSI and 1496 E-RICSI oocyte retrieval cycles were matched, and the embryologic outcomes, clinical outcomes and neonatal outcomes were compared between matched conventional ICSI and E-RICSI groups. Owing to the inclusion of partial E-RICSI in the E-RICSI group, the embryos in the E-RICSI group were divided into two subgroups according to the fertilization methods: an IVF subgroup (no fertilization took place) and an E-RICSI subgroup, and the embryologic outcomes, clinical outcomes and neonatal outcomes were compared among the conventional ICSI group, the IVF subgroup and the E-RICSI subgroup. This study was approved by the ethical committee of Tongji Hospital (No. TJ-IRB20170803).

### Clinical procedures

Each patient was subjected to an individualized controlled ovarian stimulation (COS) protocol according to their ovarian reserve and other clinical characteristics. GnRH-agonist (GnRH-a) long protocol, GnRH antagonist protocol, GnRH-a ultra-long protocol, and luteal-phase stimulation were the most common protocols in our center, and the details had been previously described (Guo *et al.*, 2021). When two leading follicles reached a mean diameter of 18 mm or three follicles reached a mean diameter of 17 mm, recombinant hCG (250 mg; Ovidrel; Merck-Serono, Geneva, Switzerland) was administered (i.m.) to trigger ovulation. Oocytes were retrieved transvaginally at 36–38 h after hCG administration.

### Laboratory procedures

The method of fertilization (IVF or ICSI) depended on the sperm concentration, motility, morphology and the history of fertilization in previous cycles, and the details had been previously described (Huang *et al.*, 2015). Briefly, for the ICSI cycles, the removal of the cumulus cells and corona radiata of the oocytes occurred at 38–40 h after hCG injection. The oocytes were briefly exposed (within 30 s) to hyaluronidase (HYASE, Vitrolife, Gothenburg, Sweden), and immediately washed in G-MOPS plus (Vitrolife) at least three times. Then, the oocytes were cultured in G1 plus medium (Vitrolife) waiting for the ICSI procedure, which was usually performed 40–43 h after hCG injection. As for the IVF cycles, the insemination was performed within 4 h after retrieval. After a 3-h co-incubation of oocyte and spermatozoa, the oocytes were released from the fertilization medium. Then, 1 h later, the granular cells were removed by pipettes for the second polar body check. If fewer than 50% of the mature Metaphase II oocytes (MII) released a second polar body at 6 h after the initial insemination, RICSI was performed immediately on the oocytes with only one polar body observed.

All of the embryos were checked on the morning of Days 1–3 after oocyte retrieval. On Day 3, cell number, cell fragments, and symmetry of blastomeres were evaluated, and normally fertilized embryos with ≥6 cells, uniform or slightly nonuniform cell size, and <20% cell fragments were classified as high-quality cleavage embryos. Only high-quality cleavage embryos could be considered for transfer or freezing. On Days 5 and 6, the blastocysts were evaluated according to the Gardner scoring system (Gardner *et al.*, 2000). Blastocysts ≥3BC were classified as viable blastocysts and those ≥3BB were classified as high-quality blastocysts.

On Day 3, one best-quality embryo was chosen for transfer and the remaining embryos were cultured to the blastocyst stage. However, for patients with zero or more than six high-quality cleavage embryos, single blastocyst transfer was recommended. If the patients had contraindications for fresh ET and obtained ≥1 high-quality cleavage embryo on Day 3, one high-quality cleavage embryo was frozen, and the rest were cultured to blastocyst stage. Otherwise, blastocyst culture was recommended for all embryos.

### Follow-up and measure outcomes

Serum hCG was tested at 2 weeks after ET, and ultrasound examinations were performed at 4 weeks after ET. At 1–3 months after the expected date of confinement, the patients were contacted by telephone to gather the obstetrical and neonatal outcomes, including the obstetrical complications, date of birth, sex of neonates, birthweight (BW), any birth defects and the health conditions of puerpera and neonates.

The measure outcomes in this study consisted of embryologic outcomes, clinical outcomes and neonatal outcomes, and the primary outcome was CLBR.

The definitions of embryologic outcomes were the same as the Vienna consensus (ESHRE Special Interest Group of Embryology and Alpha Scientists in Reproductive Medicine, 2017). Of note is that the denominator of two pronucleus (2PN), one pronucleus (1PN), and polyspermy (multi-PN) rate in the E-RICSI group, and IVF subgroup was the number of MII at the moment that E-RICSI was performed, including those not undergoing E-RICSI. As regards clinical outcomes, clinical pregnancy was defined as the presence of one or more gestational sacs with fetal heart activity in the uterus on ultrasound; ectopic pregnancy was the presence of one or more gestational sacs outside the uterus confirmed by sonography or laparoscopy, and miscarriage was the complete loss of the fetus before 28 weeks gestation. Cumulative clinical pregnancy rate (CCPR)/CLBR was presented as the percentage of women with clinical pregnancies/live births after one oocyte retrieval over 2 years and only the first time was counted when women had multiple clinical pregnancies/live births.

As for the neonatal outcomes, preterm birth (PB) was birth before 37 completed weeks of gestation; low BW (LBW) was a weight of <2500 g at birth, and macrosomia was a weight of ≥4000 g at birth.

### Statistical analysis

Statistical analysis was performed using Statistical Package for Social Sciences (SPSS) software version 25.0 (IBM, Almond, NY, USA). For continuous variables, a test of normality was conducted before analysis. Normally distributed data were presented as mean ± SD, and an independent Student’s *t*-test or one-way ANOVA was used for comparison, and the least square difference test was applied for *post hoc* comparisons. Non-normally distributed data were presented as medians (interquartile range), and the Mann–Whitney test was used for comparison. For categorical variables, the data were presented as percentages, the chi-squared test was used for comparison, and the *P*-values in the post hoc comparisons were adjusted using Boferroni’s method. When comparing the clinical outcomes and neonatal outcomes between conventional ICSI cycles and those transferring E-RICSI embryos, potential confounder adjustment was performed using logistic regression analysis. The regression models for clinical outcomes included maternal age, BMI, endometrial thickness, COS protocol (for fresh cycles)/endometrial preparation protocol (for frozen cycles), infertility factors, antral follicle count (AFC), number of oocytes retrieved, number of transferred embryos, and stage of transferred embryos (cleavage or blastocyst), while all neonatal outcome models also accounted for maternal age, BMI, etiology of infertility, COS protocol (for fresh cycles)/endometrial preparation protocol (for frozen cycles), infertility factors, stage of transferred embryos, number of newborns, and fetal sex. *P*-values <0.05 were considered statistically significant.

PSM was performed using the SPSS ‘propensity scoring’ function. The probability of E-RICSI treatment (the propensity score) was estimated for each patient using a logistic regression model that took maternal age, BMI, etiology of infertility (primary or secondary infertility), AFC, basal FSH level, number of retrieved oocytes, and COS protocol as covariates. A higher propensity score indicated a higher probability of undergoing E-RICSI treatment. E-RICSI and conventional ICSI patients were matched one-to-one with a maximum propensity score difference of 0.01 within each matched pair.

## Results

### Propensity score matching

As shown in Table 1, most of the baseline characteristics were significantly different between conventional ICSI and E-RICSI groups before PSM. After PSM, 1496 conventional ICSI oocyte retrieval cycles and 1496 E-RICSI oocyte retrieval cycles were matched, and no differences in baseline variables were observed between the groups, except for the COS protocols and infertility factors.

**Table 1.** Comparison of baseline characteristics of patients in the conventional ICSI and E-RICSI groups.

	Before PSM		*P*	After PSM		*P*
	ICSI group (N = 6279)	E-RICSI group (N = 1500)		ICSI group (N = 1496)	E-RICSI group (N = 1496)	
Maternal age (years)	32.5 ± 5.3	30.9 ± 4.6	<0.001	30.9 ± 4.6	30.9 ± 4.6	0.749
BMI (kg/m^2^)	22.0 ± 2.9	21.9 ± 3.0	0.182	22.0 ± 3.1	21.9 ± 3.0	0.477
Infertility						
Primary infertility	4165 (66.3%)	1078 (71.9%)	<0.001	1069 (71.5%)	1074 (71.8%)	0.839
Secondary infertility	2114 (33.7%)	422 (28.1%)		427 (28.5%)	422 (28.2%)	
Infertility duration (years)	3 (2, 5)	3 (2, 5)	0.033	3 (2, 5)	3 (2, 5)	0.199
Infertility factors, n (%)			<0.001			<0.001
Ovulation dysfunction	913 (14.5%)	179 (11.9%)		143 (9.6%)	176 (11.8%)	
Tubal factor	960 (15.3%)	329 (21.9%)		270 (18.0%)	332 (22.2%)	
Endometriosis	127 (2.0%)	43 (2.9%)		35 (2.3%)	43 (2.9%)	
Male factor	1076 (17.1%)	229 (15.3%)		321 (21.5%)	241 (16.1%)	
Unexplained infertility	528 (8.4%)	267 (17.8%)		143 (9.6%)	268 (17.9%)	
Multiple female factor	1039 (16.5%)	175 (11.7%)		183 (12.2%)	170 (11.4%)	
Both female and male	1636 (26.1%)	278 (18.5%)		401 (26.8%)	266 (17.8%)	
AFC	11.6 ± 7.2	14.0 ± 7.4	<0.001	13.5 ± 7.3	13.9 ± 7.2	0.188
COS protocols			<0.001			<0.001
Luteal-phase stimulation	997 (15.9%)	100 (6.7%)		183 (12.2%)	100 (6.7%)	
GnRH-a ultra-long protocol	1729 (27.5%)	680 (45.3%)		618 (41.3%)	678 (45.3%)	
GnRH antagonist protocol	1937 (30.8%)	401 (26.7%)		413 (27.6%)	399 (26.7%)	
GnRH-a long protocol	1189 (18.9%)	288 (19.2%)		261 (17.4%)	288 (19.3%)	
Others	427 (6.8%)	31 (2.1%)		21 (1.4%)	31 (2.1%)	
Duration of stimulation	9.9 ± 2.1	10.3 ± 2.1	<0.001	10.4 ± 2.1	10.3 ± 2.1	0.602
Gn (IU)	2532.1 ± 952.2	2459.5 ± 918.0	0.008	2505.4 ± 889.2	2487.2 ± 892.6	0.577
FSH levels (mIU/ml)	8.1 ± 3.4	7.6 ± 2.3	<0.001	7.7 ± 2.5	7.6 ± 2.3	0.129
No. of oocytes retrieved	10.7 ± 7.0	12.6 ± 6.5	<0.001	12.2 ± 7.0	12.6 ± 6.5	0.122

Note: Infertility duration is expressed as median (interQuartile range), other data are expressed as mean ± SD or percentage.

E-RICSI, early rescue ICSI; PSM, propensity score matching; AFC, antral follicle count; COS, controlled ovarian hyperstimulation; GnRH-a: GnRH-agonist; Gn: gonadotrophin.

### Embryologic outcomes

As shown in Table 2, the 2PN rate, blastocyst formation rate, and viable blastocyst formation rate of the E-RICSI group were significantly lower than the conventional ICSI group, while the MII rate and multi-PN rate of the E-RICSI group were higher than the conventional ICSI group, and the 1PN rate, normal cleavage rate, D3 high-quality cleavage embryo rate and high-quality blastocyst rate were comparable between groups. However, when considering the absolute number, the number of 2PN embryos, normal cleavage embryos, and frozen embryos were similar between conventional ICSI and E-RICSI groups, and the number of MII embryos, 1PN embryos and multi-PN embryos in the E-RICSI group were significantly higher than in the conventional ICSI group (1PN: 0.35 ± 0.64 versus 0.29 ± 0.58, *P* = 0.009; multi-PN: 0.36 ± 0.75 versus 0.15 ± 0.53, *P* < 0.001).

**Table 2.** Comparison of embryologic outcomes and cumulative pregnancy outcomes between the conventional ICSI and E-RICSI groups.

	ICSI group (N = 1496)	E-RICSI group (N = 1496)		
		Total	IVF subgroup	E-RICSI subgroup
MII rate	4537/18 233 (79.7%)	15 599/18 803 (83.0%)*		
2PN rate	10 312/14 537 (70.9%)^a^	10 542/15 599 (67.6%)*	1553/3222 (48.2%)^b^	8989/12 377 (72.6%)^c^
1PN rate	432/14 537 (3.0%)	520/15 599 (3.3%)	107/3222 (3.3%)	413/12 377 (3.3%)
Multi-PN rate	225/14 537 (1.5%)^a^	532/15 599 (3.4%)*	79/3222 (2.5%)^b^	453/12 377 (3.7%)^c^
Normal cleavage rate	10 006/10 312 (97.0%)	10 188/10 542 (96.6%)	1516/1553 (97.6%)	8672/8989 (96.5%)
D3 high-quality cleavage embryo rate	4336/10 006 (43.4%)	4490/10 188 (44.1%)	706/1516 (46.6%)	3784/8672 (43.6%)
Blastocyst formation rate	5220/8184 (63.8%)^a^	4934/8172 (60.4%)*	720/1191 (60.5%)^a,b^	4214/6981 (60.4%)^b^
Viable blastocyst formation rate	3208/8184 (39.2%)^a^	3023/8172 (37.0%)*	439/1191 (36.9%)^a,b^	2584/6981 (37.0%)^b^
High-quality blastocyst rate	1797/5220 (34.4%)	1722/4934 (34.9%)	260/720 (36.1%)	1462/4214 (34.7%)
No. of MII	9.7 ± 5.8	10.4 ± 5.6*		
No. of 2PN	6.9 ± 4.7	7.1 ± 4.3		
No. of 1PN	0.29 ± 0.58	0.35 ± 0.64*		
No. of multi-PN	0.15 ± 0.54	0.36 ± 0.75*		
No. of normal cleavage embryos	6.7 ± 4.6	6.8 ± 4.2		
No. of frozen embryos	2 (1, 4)	2 (1,3)		
Cumulative clinical pregnancy rate	941/1496 (62.9%)	972/1496 (65.0%)		
Cumulative live birth rate	832/1496 (55.6%)	878/1496 (58.7%)		

Note: No. of frozen embryos is expressed as median (interquartile range), other data are expressed as mean ± SD or percentage.

E-RICSI, early rescue ICSI; MII, metaphase II oocytes; PN, pronucleus.

a,b,c Values with different superscript letters indicate significant differences between different subgroups.

* *P*-value <0.05 compared to conventional ICSI group.

When considering the IVF embryos and E-RICSI embryos independently, it was found that the 2PN rate of the conventional ICSI group was significantly lower than the E-RICSI subgroup but higher than the IVF subgroup, whereas the blastocyst formation rate and viable blastocyst formation rate were higher than E-RICSI embryos but comparable with IVF embryos. In addition, the multi-PN rate of the IVF subgroup was higher than conventional ICSI but lower than the E-RICSI subgroup. Furthermore, the 1PN rate, normal cleavage rate, D3 high-quality cleavage embryo rate, and high-quality blastocyst rate were all similar among groups.

### Clinical outcomes

As shown in Fig. 1, after oocyte retrieval, 902 conventional ICSI cycles and 970 E-RICSI cycles underwent fresh ET. Among the 970 E-RICSI cycles, 215 transferred embryos fertilized by IVF, 89 by IVF and E-RICSI, and 666 by E-RICSI. Cycles that transferred both IVF and E-RICSI embryos were excluded in the subsequent analysis because it was difficult to track the source of embryos when patients became pregnant. The baseline characteristics are shown in Supplementary Table S1 and the clinical outcomes in Tables 3 and 4. We found that the E-RICSI subgroup had a significantly lower rate of single-ET and a higher rate of double-embryos compared to the IVF subgroup and conventional ICSI group, but the stage of transferred embryos was similar among groups. As a result, the implantation rate in the IVF subgroup was significantly higher than the E-RICSI subgroup but comparable with the conventional ICSI group, and the clinical pregnancy rate, ectopic pregnancy, miscarriage rate, and live birth rate were comparable among groups. The results were stable after adjusting for maternal age, maternal BMI, endometrial thickness, COS protocols, infertility factors, AFC, number of oocytes retrieved, number of transferred embryos, and stage of transferred embryos. Only the implantation rate in the IVF subgroup was significantly different when compared to the E-RICSI subgroup (adjusted OR and 95% CI: 1.413 [1.068, 1.871], *P* = 0.016).

**Table 3.** Comparison of clinical outcomes among the conventional ICSI group, IVF subgroup and E-RICSI subgroup.

		Fresh cycles			Frozen cycles		
		ICSI group	IVF subgroup	E-RICSI subgroup	ICSI group	IVF subgroup	E-RICSI subgroup
No. of ET cycles		902	215	666	1053	158	841
No. of embryos transferred							
1		663 (73.5%)^a^	163 (75.8%)^a^	447 (67.1%)^b^	735 (69.8%)^a^	128 (81.0%)^b^	638 (75.9%)^b^
2		239 (26.5%)	52 (24.2%)	219 (32.9%)	318 (30.2%)	30 (19.0%)	203 (24.1%)
Stage of embryos transferred							
Cleavage stage		820 (90.9%)	191 (88.8%)	606 (91.0%)	226 (21.5%)	33 (20.9%)	181 (21.5%)
Blastocyst stage		82 (9.1%)	24 (11.2%)	60 (9.0%)	827 (78.5%)	125 (79.1%)	660 (78.5%)
Implantation rate	535/1141 (46.9%)^a,b^		142/267 (53.2%)^a^	379/885 (42.8%)^b^	678/1371 (49.5%)	97/188 (51.6%)	534/1044 (51.1%)
Clinical pregnancy rate		463/902 (51.3%)	118/215 (54.9%)	320/666 (48.0%)	557/1053 (52.9%)	87/158 (55.1%)	455/841 (54.1%)
Ectopic pregnancy rate		13/902 (1.4%)	2/215 (0.9%)	4/666 (0.6%)	5/1053 (0.5%)	0 (0)	1/841 (0.1%)
Miscarriage rate		77/463 (16.6%)	13/118 (11.0%)	40/320 (12.5%)	93/557 (16.7%)	17/87 (19.5%)	88/455 (19.3%)
Live birth rate		387/902 (42.9%)	104/215 (48.4%)	279/666 (41.9%)	464/1053 (44.1%)	70/158 (44.3%)	367/841 (43.6%)

E-RICSI, early rescue ICSI; ET, embryo transfer.

a,b Values with different superscript letters indicate significant differences between different subgroups.

**Table 4.** Logistic regression analysis of different fertilization types on clinical and neonatal outcomes using E-RICSI subgroup as reference.

	Fresh cycles				Frozen cycles			
	IVF subgroup Adjusted OR (95% CI)	*P*	Conventional ICSI group Adjusted OR (95% CI)	*P*	IVF subgroup Adjusted OR (95% CI)	*P*	Conventional ICSI group Adjusted OR (95% CI)	*P*
Implantation^a^	1.413 (1.068, 1.871)	0.016	1.161 (0.969, 1.391)	0.106	0.898 (0.647, 1.247)	0.522	0.938 (0.791, 1.114)	0.467
Clinical pregnancy^a^	1.304 (0.952, 1.786)	0.099	1.148 (0.935, 1.409)	0.187	0.957 (0.663, 1.383)	0.817	0.910 (0.749, 1.107)	0.345
Ectopic pregnancy^a^	1.767 (0.316, 9.864)	0.517	2.404 (0.771, 7.495)	0.131	NA		4.384 (0.504, 38.157)	0.181
Miscarriage^a^	0.873 (0.446, 1.710)	0.693	1.402 (0.923, 2.129)	0.113	1.101 (0.611, 1.985)	0.750	0.860 (0.618, 1.197)	0.372
Live birth^a^	1.285 (0.937, 1.761)	0.119	1.044 (0.848, 1.285)	0.685	0.941 (0.654, 1.355)	0.745	0.983 (0.809, 1.195)	0.372
Cesarean section^b^	0.737 (0.426, 1.274)	0.270	0.928 (0.633, 1.362)	0.704	1.446 (0.618, 3.384)	0.396	0.974 (0.636, 1.491)	0.902
Sex (male)^b^	1.224 (0.801,1.870)	0.350	1.092 (0.815, 1.462)	0.556	0.840 (0.512, 1.377)	0.489	0.941 (0.725, 1.221)	0.646
Preterm birth (<37 weeks)^b^	1.002 (0.528, 1.902)	0.996	0.980 (0.619, 1.553)	0.933	1.378 (0.680, 2.795)	0.374	0.926 (0.639, 1.340)	0.682
LBW (<2500 g)^b^	1.278 (0.589, 2.772)	0.534	1.841 (1.040, 3.259)	0.036	0.994 (0.398, 2.480)	0.990	0.966 (0.629, 1.482)	0.874
Macrosomia (≥4000 g)^b^	1.050 (0.379, 2.908)	0.925	1.175 (0.577, 2.396)	0.656	1.739 (0.763, 3.962)	0.188	0.951 (0.552, 1.637)	0.855

E-RICSI, early rescue ICSI; OR: odds ratio; LBW: low birthweight; OR, odds ratio.

NA, not obtained due to the small sample size.

a Logistic models adjusted for maternal age, maternal BMI, endometrial thickness, COS protocols (for fresh cycles)/endometrial preparation protocols (for frozen cycles), infertility factors, antral follicle counting, number of oocytes retrieved, number of transferred embryos, and stage of transferred embryos.

b Logistic models adjusted for maternal age, maternal BMI, COS protocols (for fresh cycles)/endometrial preparation protocols (for frozen cycles), infertility factors, stage of transferred embryos, number of newborns, and fetal sex.

A total of 1058 frozen cycles were conducted in the conventional ICSI group, and five were canceled because there were no viable embryos after thawing, while 1059 frozen cycles were conducted in the E-RICSI group and one was canceled. Of these, 158 cycles transferred embryos fertilized by IVF, 59 by IVF and E-RICSI, and 842 by E-RICSI. Finally, 841 E-RICSI cycles and 158 IVF cycles were enrolled in the analysis (one cycle was canceled) and the results are shown in Tables 3 and 5 and Supplementary Table S2. The conventional ICSI group had a significantly lower rate of single-ET and a higher rate of double-embryos than the IVF and E-RICSI subgroups, but the stage of transferred embryos was similar among groups. No significant differences were observed among the IVF and E-RICSI subgroups and the conventional ICSI group on implantation rate, clinical pregnancy rate, ectopic pregnancy, miscarriage rate, and live birth rate, and the results did not alter after adjustment for maternal age, maternal BMI, endometrial thickness, endometrial preparation protocols, infertility factors, AFC, number of oocytes retrieved, number of transferred embryos, and stage of transferred embryos (Table 4).

**Table 5.** Comparision of neonatal outcomes among the conventional ICSI group, IVF subgroup, and E-RICSI subgroup.

	Fresh cycles			Frozen cycles		
	ICSI group	IVF subgroup	E-RICSI subgroup	ICSI group	IVF subgroup	E-RICSI subgroup
No. of live births	434	120	316	533	75	414
No. of newborns						
Singletons	340 (78.3%)	88 (73.3%)	243 (76.9%)	390 (73.2%)^b^	65 (86.7%)^a^	318 (76.8%)^a,b^
Twins	94 (21.7%)	32 (26.7%)	73 (23.1%)	143 (26.8%)	10 (13.3%)	96 (23.2%)
Cesarean section	349 (80.4%)	94 (78.3%)	254 (80.4%)	476 (89.3%)	68 (90.7%)	368 (88.9%)
Sex						
Male	222 (51.2%)	65 (54.2%)	154 (48.7%)	273 (51.2%)	37 (49.3%)	220 (53.1%)
Female	212 (48.8%)	55 (45.8%)	162 (51.3%)	260 (48.8%)	38 (50.7%)	194 (46.9%)
Gestational age (weeks)	38.3 ± 1.7	38.4 ± 1.9	38.5 ± 1.6	38.1 ± 1.2	38.3 ± 2.4	38.2 ± 2.1
Preterm birth (<37 weeks)	65 (15.0%)	21 (17.5%)	50 (15.8%)	119 (22.3%)	15 (20.0%)	91 (22.0%)
Birth weight (g)	3083.6 ± 585.9	3091.0 ± 585.8	3138.9 ± 528.2	3104.5 ± 639.6^b^	3303.4 ± 634.8^a^	3140.9 ± 615.4^b^
LBW (<2500 g)	62 (14.3%)	17 (14.2%)	33 (10.4%)	77 (14.4%)	7 (9.3%)	55 (13.3%)
Macrosomia (≥4000 g)	23 (5.3%)	6 (5.0%)	13 (4.1%)	33 (6.2%)	9 (12.0%)	27 (6.5%)

E-RICSI, early rescue ICSI; LBW, low birthweight.

a,b Values with different superscript letters indicate significant differences between different subgroups.

As a result, the CCPR and CLBR were both comparable between conventional ICSI and E-RICSI groups (Table 2).

### Neonatal outcomes

Only live births were included in the analysis. For fresh conventional ICSI cycles, 340 singletons and 47 pairs of twins were born, and all of them were born alive. After fresh E-RICSI-fertilized ET cycles, 243 singletons and 37 pairs of twins were born, with one twin newborn born dead. In fresh IVF-fertilized ET cycles, 88 singletons and 16 pairs of twins were born, with all born alive. As shown in Tables 4 and 5, no differences were observed for the neonatal outcomes including fetal sex, gestational age (GA), BW, rate of cesarean section, PB, LBW, and macrosomia among the conventional ICSI group and the IVF and E-RICSI subgroups. After adjusting for maternal age, maternal BMI, COS protocols, infertility factors, stage of transferred embryos, number of newborns, and fetal sex, it was found that, compared to the E-RICSI subgroup, the risk of LBW was significantly increased in the conventional ICSI group (adjusted OR and 95% Ci: 1.841 [1.040, 3.259], *P* = 0.036).

Three hundred and ninety singletons and 74 pairs of twins were born after frozen conventional ICSI cycles, and five twin newborns were stillbirths; 318 singletons and 49 pairs of twins were born after frozen E-RICSI-fertilized ET cycles, with two twin newborns born dead, while 65 singletons and 5 pairs of twins were born after frozen IVF-fertilized ET cycles and all were born alive. The proportion of singletons in the conventional ICSI group was significantly lower than the IVF subgroup, but comparable with the E-RICSI subgroup (Tables 4 and 5). The BW in the IVF subgroup was significantly heavier than the other two groups, but the LBW rate was comparable among groups. Other than that, there were no significant differences in fetal sex, rate of cesarean section, PB, LBW, and macrosomia among groups, and the results were stable after adjusting for maternal age, maternal BMI, endometrial preparation protocols, infertility factors, stage of transferred embryos, number of newborns, and fetal sex.

## Discussion

Theoretically speaking, short-time insemination combined with E-RICSI is more reliable than conventional ICSI for patients with broadline or normal semen characteristics for preventing the occurrence of TFF in IVF, considering the potential detriment to embryo development and offspring health issues associated with ICSI (Allen and Reardon, 2005; Amor and Halliday, 2008; Sandin *et al.*, 2013; Qin *et al.*, 2015). However, the limited reports on the pregnancy outcomes and safety of E-RICSI have restricted its clinical application. Short-time insemination combined with E-RICSI has been used in our center for more than 15 years, and the incidence of TFF in IVF cycles has been 1.0–1.7% in recent years, which is significantly lower than previous reports (Liu and Baker, 2000; Bhattacharya *et al.*, 2001; Krog *et al.*, 2015). To evaluate the efficacy and safety of E-RICSI, we conducted this study to compare the embryologic, clinical, and neonatal outcomes in patients with E-RICSI versus conventional ICSI. PSM was used to minimize the selection bias and a more comprehensive indicator (CLBR) was selected as the primary measure outcome. It was found that E-RICSI did not add extra risks to the clinical and neonatal outcomes compared to conventional ICSI.

This study demonstrated that the 2PN rate in the E-RICSI group was significantly lower than in the conventional ICSI group. However, when considering the absolute number, the number of 2PN embryos in the E-RICSI group was similar to the conventional ICSI group. The increased number of MII in the E-RICSI group may explain these conflicting results. Owing to the extended co-culture with cumulus cells in E-RICSI treatment, the MII rate and number of MII in the E-RICSI group are higher than the conventional ICSI group. As a result, the 2PN rate in the E-RICSI group ostensibly declines despite the number of 2PN embryos being stable. Similar results were also observed in another study (Zeng *et al.*, 2022), but the researchers ascribe the decreased 2PN rate in the E-RICSI group to oocyte aging and impaired embryo development. Our study demonstrates that E-RICSI does not impair the number of 2PN embryos, and the 2PN rate in the E-RICSI group is apparently decreased because of an increase in MII.

When we considered the IVF and E-RICSI embryos in the E-RICSI group independently, the 2PN rate in the conventional ICSI group was higher than the IVF subgroup but lower than the E-RICSI subgroup. Determining the number of polar bodies for oocytes with fragmented polar bodies is so difficult that most embryologists prefer not to perform E-RICSI on them in case of polyspermy. As a result, the 2PN rate in the IVF subgroup decreased. Interestingly, injection time in the E-RICSI subgroup is 2–5 h later than the conventional ICSI group, and its 2PN rate is higher but its blastocyst formation rate and viable blastocyst formation rate are lower. The optimal timing for ICSI has been controversial. Bárcena *et al.* (2016) reported a wide time interval between oocyte retrieval and ICSI, ranging from 1 h 25 min to 17 h 13 min, and no significant differences in fertilization rate and pregnancy outcomes were observed within that time interval. Smith *et al.* (2021) revealed that the timing of ICSI was acceptable within 2–6 h post oocyte retrieval. However, another study found that extended time intervals between oocyte retrieval and ICSI had a downstream effect on live birth rate and CLBR, and extended time intervals between oocyte retrieval and cumulus cell removal had an upstream effect on pregnancy rate, live birth rate, and CLBR (Carvalho *et al.*, 2020). Our results implied that extended co-culture with cumulus cells and delayed injection, within a certain timeframe, might accelerate the oocyte cytoplasmic maturity, thereby increasing the normal fertilization rate, but it might also lead to the aging of cellular organelles with insufficient energy supply from mitochondria, resulting in a decrease in blastocyst formation. Besides, extended co-culture with cumulus cells can also facilitate the IVM of immature oocytes, which may be associated with the decreased blastocyst formation rate in the E-RICSI subgroup. It has been reported that the blastocyst formation rate and viable blastocyst formation rates in embryos derived from IVM are significantly lower than those from standard ICSI treatment (Walls *et al.*, 2015), which is consistent with our hypothesis.

Accurate identification of the second polar body has always been a challenge in E-RICSI treatment, and the experience of embryologists in observing the second polar body and assessing the status of fertilization plays an important role. The lower 2PN rate in the IVF subgroup has implied the cautious and conservative attitude of embryologists in the process of performing the E-RICSI procedure, but the polyspermy rate and number of polyspermy embryos in the E-RICSI group were still significantly higher compared to the conventional ICSI group, and similar results were also observed when considering IVF and E-RICSI embryos independently. Although the subjective misjudgment of embryologists contributes to higher polyspermy fertilization rates, the effect of extended co-culture with cumulus cells should also be considered. Cumulus cells provide oocytes with a variety of substances, including steroid hormones, glycosaminoglycan, and nutrients, thereby promoting oocyte nuclear and cytoplasmic maturation (Wongsrikeao *et al.*, 2005). The delayed removal of cumulus cells can facilitate the IVM of immature oocytes and generate more mature oocytes compared to conventional ICSI. Some of these delayed matured oocytes are fertilized while co-incubated with sperm, but E-RICSI is performed incorrectly because the second polar bodies are not released at the time of the polar body observation, resulting in an increased polyspermy rate. On the other hand, the unfertilized oocytes, including delayed matured ones, are saved through E-RICSI, compensating for the loss caused by polyspermy. As a result, the number of 2PN embryos is comparable between the E-RICSI and conventional ICSI groups.

Notably, this study is the first to report the CLBR and the pregnancy outcomes of RICSI patients in frozen cycles. Previous substantial studies have examined the pregnancy outcomes of RICSI patients in fresh cycles, and no significant differences were found between RICSI and conventional ICSI patients in pregnancy rate and live birth rate (Chen *et al.*, 2014; Huang *et al.*, 2015; He *et al.*, 2018; Xiong *et al.*, 2020; Zeng *et al.*, 2022). However, with the advances in embryo cryopreservation technology, embryos can be well-preserved, thawed and transferred to the uterus after ovary recovery and endometrial preparation. Therefore, consideration of pregnancy outcomes in frozen cycles is also important. This study investigated the clinical outcomes of RICSI patients in both fresh and frozen cycles and found that in fresh ET cycles, the implantation rate of the E-RICSI subgroup was significantly lower than the IVF subgroup but comparable with the conventional ICSI group, while in frozen cycles, the implantation rate is comparable among groups. The other clinical outcomes were all similar among groups in both fresh and frozen cycles, further confirming the previous findings. CLBR is suggested as a comprehensive indicator that considers both fresh and frozen ET cycles, but it has not been reported in previous studies (Maheshwari *et al.*, 2015). Xiong *et al.* (2020) found a higher polyspermy rate and lower number of top-quality embryos on Day 3 in E-RICSI patients compared to conventional ICSI patients and speculated that the CPR and CLBR were reduced in the E-RICSI group. However, in their study, only embryos fertilized by E-RICSI were counted in the analysis while E-RICSI was not performed on all embryos. The present study included patients with partly E-RICSI treatment and counted the IVF-fertilized embryos in the analysis, finding that the increased implantation rate of fresh IVF embryos in the E-RICSI group compensated for the loss from the decreased viable blastocyst formation rate, and ultimately, the CCPR and CLBR were comparable between the E-RICSI and conventional ICSI groups. Our results demonstrated that E-RICSI had no detrimental effects on the clinical outcomes.

In our study, we also found that the LBW rate of the conventional ICSI group was higher than the E-RICSI group among fresh cycles after adjusting for confounders. Otherwise, neonates fertilized by E-RICSI showed no difference in neonatal outcomes compared to those (fertilised by) IVF and conventional ICSI, regardless of whether they were born after fresh cycles or frozen cycles. Thus, the results of our study support the safety of E-RICSI treatment for neonates, but further research is needed.

However, this study also has its limitations. First, this is a retrospective study. Although we employ PSM and exclude patients with severe oligospermia or azoospermia to decrease the selection bias, it is still challenging to avoid potential bias from unbalanced semen quality. Also, the infertility factors and COS protocols are different between the E-RICSI and conventional ICSI groups, which may be an important bias in this study. Second, the PSM is only performed for the initial selection. For the patients comparing pregnancy outcomes per transfer and neonatal outcomes, the advantage of PSM is no longer valid. Thus, logistic regression is applied to adjust for confounders. Finally, the sample size is small owing to the low frequency of E-RICSI incidence, and the follow-up period is also short. More large-scale randomized controlled trials with long-term follow-up are needed to further validate the safety and efficacy of E-RICSI.

## Conclusion

To conclude, our results indicate that E-RICSI has no detrimental effects on clinical and neonatal outcomes compared to conventional ICSI. Therefore, short-term insemination combined with E-RICSI may be a safe and effective method to prevent the occurrence of TFF in IVF, and patients with normal or borderline sperm could be encouraged to try short-term insemination IVF first.

## Supplementary Material

hoad046_Supplementary_Data

## Data Availability

The data underlying this article cannot be shared publicly due to the privacy of individuals that participated in the study. The data will be shared on reasonable request to the corresponding author.
