# Supplementary material for: Cumulative live birth rate and neonatal outcomes after early rescue ICSI: a propensity score matching analysis
Source: Hum Reprod Open. 2023 Nov 23;2023(4):hoad046. doi: 10.1093/hropen/hoad046 (PMC10719215; doi:10.1093/hropen/hoad046)
Supplement: hoad046_Supplementary_Data [file hoad046_supplementary_data.docx]

**Supplementary Table S1** The baseline characteristics of patients underwent fresh embryo transfer among the conventional ICSI group, IVF subgroup and E-RICSI subgroup.

|  | 1CS1 group | 1VF subgroup | E-RICSI subgroup | P |
| --- | --- | --- | --- | --- |
| No. of ET cycles | 902 | 215 | 666 |  |
| Maternal age (years) | 30.4 ± 4.0 | 30.1 ± 3.6 | 30.4 ± 3.9 | 0.630 |
| BMI (kg/m^2^) | 22.0 ± 3.0 | 21.9 ± 3.1 | 21.9 ± 2.9 | 0.884 |
| Infertility |  |  |  | 0.871 |
| Primary infertility | 669(74.2%) | 161(74.9%) | 488(73.3%) |  |
| Secondary infertility | 233(25.8%) | 54(25.1%) | 178(26.7%) |  |
| Infertility duration (years) | 3 (2, 5) | 3 (2, 5) | 3 (2, 5) | 0.912 |
| Infertility factors, % (n) |  |  |  | <0.001 |
| Ovulation dysfunction | 81 (9.0%) | 27 (12.6%) | 68 (10.2%) |  |
| Tubal factor | 170 (18.8%)^b^ | 49 (22.8%)^a,b^ | 167 (25.1%)^a^ |  |
| Endometriosis | 19 (2.1%) | 5 (2.3%) | 22 (3.3%) |  |
| Male factor | 229 (25.4%)^b^ | 44 (20.5%)^a,b^ | 13 (17.0%)^a^ |  |
| Unexplained infertility | 93 (10.3%)^b^ | 40 (18.6%)^a^ | 123 (18.5%)^a^ |  |
| Multiple female factors | 74 (8.2%) | 16 (7.4%) | 63 (9.5%) |  |
| Both female and male | 236 (26.2%)^b^ | 34 (15.8%)^a^ | 110 (16.5%)^a^ |  |
| AFC | 14.2 ± 6.8 | 14.7 ± 6.9 | 13.9 ± 6.7 | 0.297 |
| COS protocols |  |  |  | 0.134 |
| GnRH-a ultra-long protocol | 459 (50.9%) | 126 (58.6%) | 317 (47.6%) |  |
| GnRH antagonist protocol | 267 (29.6%) | 58 (27.0%) | 213 (32.0%) |  |
| GnRH-a long protocol | 169 (18.7%) | 29 (13.5%) | 127 (19.1%) |  |
| Others | 7 (0.9%) | 2 (0.9%) | 9 (1.4%) |  |
| Duration of stimulation | 10.5 ± 2.2 | 10.5 ± 1.8 | 10.4 ± 2.1 | 0.266 |
| Gn (IU) | 2474.9 ± 889.6 | 2466.5 ± 848.0 | 2491.9 ± 829.6 | 0.900 |
| FSH levels (mIU/ml) | 7.5 ± 2.0 | 7.6 ± 2.3 | 7.6 ± 2.1 | 0.469 |
| No. of oocytes retrieved | 11.4 ± 5.0^b^ | 12.4 ± 4.7^a^ | 11.5 ± 4.7^b^ | 0.029 |
| Endometrial thickness | 11.9 ± 2.5 | 12.0 ± 2.6 | 11.9 ± 2.4 | 0.721 |

Infertility duration is expressed as median (interQuartile rang), other data are expressed as mean ± SD or percentage

E-RICSI, early rescue ICSI; ET, embryo transfer; AFC, Antral follicle counting; COS, controlled ovarian hyperstimulation; GnRH-a: GnRH-agonist; Gn: gonadotrophin.

^a,b,c^Values with different superscript letters indicate significant differences between different subgroups.

**Supplementary Table S2** The baseline characteristics of patients underwent frozen embryo transfer among the conventional ICSI group, IVF subgroup and E-RICSI subgroup.

|  | 1CS1 group | 1VF subgroup | E-RICSI subgroup | P |
| --- | --- | --- | --- | --- |
| No. of ET cycles | 1053 | 158 | 841 |  |
| Maternal age (years) | 31.7 ± 4.9 | 30.9 ± 4.8 | 31.5 ± 4.8 | 0.111 |
| BMI (kg/m^2^) | 21.9 ± 3.1 | 22.0 ± 3.0 | 21.8 ± 3.1 | 0.635 |
| Infertility |  |  |  |  |
| Primary infertility | 742 (70.5%) | 113 (71.5%) | 584 (69.4%) | 0.822 |
| Secondary infertility | 311 (29.5%) | 45 (28.5%) | 257 (30.6%) |  |
| Infertility duration (years) | 3 (2, 5) | 3 (2, 5) | 3 (2, 5) | 0.073 |
| Infertility factors, % (n) |  |  |  | <0.001 |
| Ovulation dysfunction | 79 (7.5%)^b^ | 20 (12.7%)^ab^ | 92 (10.9%)^a^ |  |
| Tubal factor | 204 (19.4%)^b^ | 44 (27.8%)^a^ | 182 (21.6%)^a.b^ |  |
| Endometriosis | 26 (2.5%) | 4 (2.5%) | 27 (3.2%) |  |
| Male factor | 217 (20.6%) | 22 (13.9%) | 149 (17.7%) |  |
| Unexplained infertility | 98 (9.3%)^b^ | 25 (15.8%)^a^ | 153 (18.2%)^a^ |  |
| Multiple female factors | 152 (14.4%)^b^ | 21 (13.3%)^ab^ | 89 (10.6%)^a^ |  |
| Both female and male | 277 (26.3%)^b^ | 22 (13.9%)^a^ | 149 (17.7%)^a^ |  |
| AFC | 13.8 ± 7.3^b^ | 16.2 ± 7.7^a^ | 13.8 ± 7.3^b^ | 0.001 |
| Endometrial preparation protocols |  |  |  | 0.153 |
| Programmed cycles | 833 (79.1%) | 131 (82.9%) | 702 (83.5%) |  |
| Natural cycles | 52 (4.9%) | 6 (3.8%) | 40 (4.8%) |  |
| Stimulated cycles | 145 (13.8%) | 16 (10.1%) | 81 (9.6%) |  |
| Others | 23 (2.2%) | 5 (3.2%） | 18 (2.1%) |  |
| FSH levels (mIU/ml) | 7.7 ± 2.5^b^ | 7.2 ± 2.2^a^ | 7.4 ± 2.1^a^ | 0.021 |
| No. of oocytes retrieved | 143 ± 7.9^b^ | 16.1 ± 7.9^a^ | 14.0 ± 7.1^b^ | 0.006 |
| Endometrial thickness | 9.4 ± 1.5 | 9.4 ± 1.7 | 9.4 ± 1.5 | 0.894 |

Infertility duration is expressed as median (interQuartile rang), other data are expressed as mean ± SD or percentage

E-RICSI, early rescue ICSI; ET, embryo transfer; AFC, antral follicle counting.

^a,b,c^Values with different superscript letters indicate significant differences between different subgroups.
